# Characterization of acute ischemia‐related physiological responses associated with remote ischemic preconditioning: a randomized controlled, crossover human study

**DOI:** 10.14814/phy2.12200

**Published:** 2014-11-20

**Authors:** Vikram Sharma, Brian Cunniffe, Amit P. Verma, Marco Cardinale, Derek Yellon

**Affiliations:** 1 The Hatter Cardiovascular Institute, UCL, London, UK; 2 Department of Internal Medicine, Cleveland Clinic, Cleveland, Ohio, USA; 3 English institute of Sport, Marlow, UK; 4 Institute of Sport, Exercise and Health, UCL, London, UK; 5 University College London, London, UK; 6 Aspire Academy, Doha, Qatar; 7 Department of Computer Science, UCL, London, UK

**Keywords:** Characterization, cuff inflation pressure, remote ischemic preconditioning, tolerability

## Abstract

Remote Ischemic Preconditioning (RIPC) is emerging as a new noninvasive intervention that has the potential to protect a number of organs against ischemia–reperfusion (IR) injury. The standard protocols normally used to deliver RIPC involve a number of cycles of inflation of a blood pressure (BP) cuff on the arm and/or leg to an inflation pressure of 200 mmHg followed by cuff deflation for a short period of time. There is little evidence to support what limb (upper or lower) or cuff inflation pressures are most effective to deliver this intervention without causing undue discomfort/pain in nonanesthetized humans. In this preliminary study, a dose–response assessment was performed using a range of cuff inflation pressures (140, 160, and 180 mmHg) to induce limb ischemia in upper and lower limbs. Physiological changes in the occluded limb and any pain/discomfort associated with RIPC with each cuff inflation pressure were determined. Results showed that ischemia can be induced in the upper limb at much lower cuff inflation pressures compared with the standard 200 mmHg pressure generally used for RIPC, provided the cuff inflation pressure is ~30 mmHg higher than the resting systolic BP. In the lower limb, a higher inflation pressure, (~55 mmHg > resting systolic BP), is required to induce ischemia. Cyclical changes in capillary blood O2, CO2, and lactate levels during the RIPC stimulus were observed. RIPC at higher cuff inflation pressures of 160 and 180 mmHg was better tolerated in the upper limb. In summary, limb ischemia for RIPC can be more easily induced at lower pressures and is much better tolerated in the upper limb in young healthy individuals. However, whether benefits of RIPC can also be derived with protocols delivered to the upper limb using lower cuff inflation pressures and with lesser discomfort compared to the lower limb, remains to be investigated.

## Abstract (precis)

Remote Ischemic Preconditioning (RIPC) is emerging as a new noninvasive intervention that has the potential to protect a number of organs against ischemia‐reperfusion (IR) injury. Currently, there is little evidence to support what limb (upper or lower) or cuff inflation pressures are most effective to deliver this intervention without causing undue discomfort/pain in nonanesthetized humans. In this study we have demonstrated that limb ischemia for RIPC can be more easily induced at lower pressures and is much better tolerated in the upper limb compared with the lower limb, in young healthy individuals.

## Introduction

Remote ischemic preconditioning (RIPC) refers to the ability of episode/s of sublethal ischemia–reperfusion (IR) in an organ/muscle to confer protection against subsequent lethal IR injury in another or distal organ (Hausenloy and Yellon 2008). It was first described by Przyklenk et al. (1993), who showed that sublethal ischemia in one vascular bed in the heart was able to protect the myocardial tissue in another vascular bed subsequently exposed to lethal IR. The authors referred to this phenomenon as “preconditioning at a distance”(Przyklenk et al. 1993). Since then, RIPC has been shown to protect a variety of organs such as the heart, kidneys, and brain against lethal ischemia–reperfusion when the lethal injury was preceded by sublethal ischemia in a remote organ (Ali et al. 2007; Zhao et al. 2007; Lazaris et al. 2009; Jensen et al. 2011; Przyklenk and Whittaker 2011; Xu et al. 2011; Alreja et al. 2012; Xie et al. 2012). Clinical studies assessing the benefits of RIPC in the setting of myocardial ischemia–reperfusion (such as in patients undergoing coronary artery bypass grafting (CABG) and/or heart valve surgery) have shown reduction in the size of myocardial infarct sustained after IR injury with RIPC, however, these have been relatively small scale studies (Venugopal et al. 2009; Pilcher et al. 2012; Xie et al. 2012). Conversely, other clinical studies have failed to reproduce these suggested RIPC cardioprotective effects (Rahman et al. 2010; Lomivorotov et al. 2012; Young et al. 2012). Very recently, larger studies and multicentre trials have started to evaluate the clinical effectiveness of RIPC in mitigating ischemia–reperfusion injury associated with CABG surgery (Hausenloy et al. 2012; Thielmann et al. 2013). Therefore, the clinical benefits of RIPC are by no means fully established and results from large randomized controlled trials are awaited to clarify its usefulness in patients. Interestingly, this phenomenon has also been looked at as a means of improving performance in highly trained athletes (de Groot et al. 2010; Jean‐St‐Michel et al. 2011). Using limb ischemia, induced in a manner similar to the RIPC protocols described earlier, has been beneficial in optimizing performance in a small number of studies in athletes (de Groot et al. 2010; Jean‐St‐Michel et al. 2011; Bailey et al. 2012a,b; Kjeld et al. 2014).

The mechanistic pathways through which the protective signal is transmitted from a remote organ to the organ/s targeted by RIPC are still being investigated. Two important routes through which this relay may occur are the humoral and neural pathways (Kanoria et al. 2007; Hausenloy and Yellon 2008; Shimizu et al. 2009; Lim et al. 2010; Jean‐St‐Michel et al. 2011; Jensen et al. 2012; Mastitskaya et al. 2012; Merlocco et al. 2014). While, the former pathway involves the release of an unknown protective factor into the blood stream that is transported to the target organ, the latter pathway involves afferent nerve activation and the subsequent transmission of the protective signal through efferent neural pathways (Shimizu et al. 2009; Lim et al. 2010; Jean‐St‐Michel et al. 2011; Jensen et al. 2012; Mastitskaya et al. 2012; Merlocco et al. 2014). Both, humoral and neural, pathways appear to be capable of conferring the benefits of RIPC to the target organ (Hausenloy and Yellon 2008; Shimizu et al. 2009; Lim et al. 2010; Jean‐St‐Michel et al. 2011; Mastitskaya et al. 2012; Merlocco et al. 2014). It remains unclear whether both the induction of limb ischemia as well as the activation of an afferent neural pathway are together required for the benefits seen with RIPC. Though a number of humoral factors have been proposed to mediate the protective effects of RIPC against lethal IR injury in a remote organ, investigators have so far been unable to identify the specific humoral factor responsible for this protection, the triggering mechanisms that lead to release of the proposed humoral mediator as well the mechanisms through which this protection is delivered to the target organs. The lack of a clearly identifiable marker to confirm the sufficiency of RIPC stimulus currently makes it difficult to predict the ideal cuff inflation pressure and the duration of cuff inflation that needs to be applied to the upper and/or lower limbs to definitively induce the neural and humoral components of this protective pathway.

In the majority of the studies evaluating the benefits of RIPC, one or more limb(s) were made ischemic for a short period of time by vascular occlusion through inflating and deflating a blood pressure cuff on the limb(s). Typically, 3–4 cycles of cuff inflation/deflation have been used, (involving 5 min of inflation–deflation) using a cuff inflation pressure of 200 mmHg (Kharbanda et al. 2001, 2002, 2006; Venugopal et al. 2009; Botker et al. 2010; Rahman et al. 2010; Heusch et al. 2012; Lomivorotov et al. 2012; Xie et al. 2012; Young et al. 2012). In children, RIPC has also been applied in the lower limb by using a cuff inflation pressure that was 15 mm of Hg above the resting systolic pressure (Cheung et al. 2006). These RIPC protocols that have been employed in various studies assessing cardiac protection and exercise performance have been based on conventional practice rather than empirical evidence. To our knowledge, there are few (if any) studies showing the optimal cuff inflation pressures that can be effectively used to induce protection against IR injury in the target organs, with particular reference to a potential dose–response relationship of different cuff inflation pressures to limb ischemia.

Also, there is limited data on how well the various RIPC protocols may be tolerated by nonanesthetized individuals. This is important to evaluate as the tolerability of this interventional may be a crucial factor in its implementation in clinical practice, particularly when this is applied to nonanesthetized subjects.

Considering the potential benefits of this noninvasive intervention in protecting a number of organs against ischemia–reperfusion injury and the emerging use of this technique in exercise physiology, it is important to establish tolerability and effectiveness of typical RIPC protocols on the cardiovascular system to guide future practice. Therefore, the aim of this preliminary study was to analyze the dose–response relationship of limb ischemia and other physiological responses in the limb to various cuff inflation pressures used to deliver RIPC in the upper and lower limb as well as to assess the tolerability of the different cuff inflation pressures used.

## Methods

All procedures were carried out with ethical approval at The Hatter Cardiovascular Institute, UCL (REC Ref. number 13/LO/0222).Six healthy male volunteers (age 31 ± 6.5 years; mean ± SD) with no known medical problems participated in this study after signing an informed consent. None of the volunteers were on any regular medications. The study was a randomized controlled crossover design.

### Protocol

**Figure 1.**
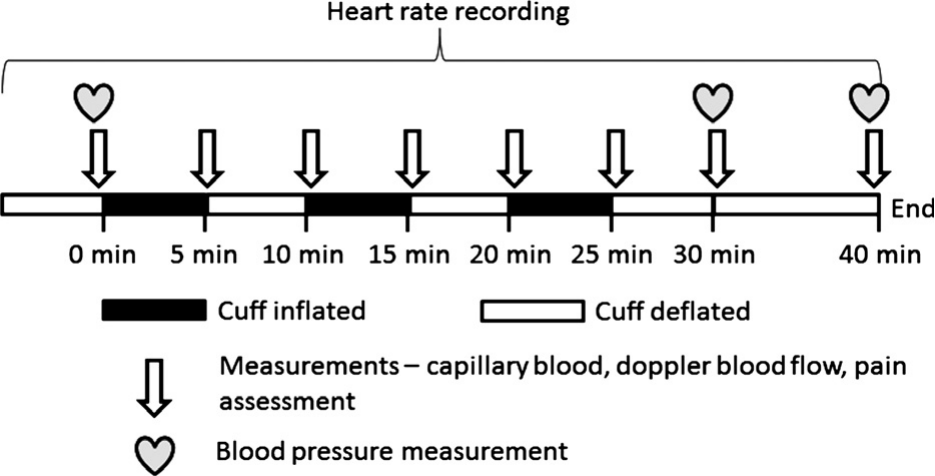
Schematic diagram describing the study protocol.

Upon entry to the lab, subjects were asked to rest in a supine position for 10 min before measuring resting blood pressure. Blood pressure (BP) was measured on the right arm using an automatic BP monitor (Omron M2 Basic, Omron Healthcare, Muko, Kyoto, Japan). Three readings were obtained and the average values were used for statistical analysis. Additional BP measures were also taken at the end of the study protocol and 10 min post each RIPC protocol (For a schematic diagram of the study design see Fig. 1). Laboratory temperature was maintained at 21°C throughout the study and all measurements were carried out between 0900 and 1700 h.

The study utilized a randomized controlled, crossover design. RIPC was administered to the right upper limb or the right lower limb using cuff inflation pressures of 140, 160, or 180 mmHg, respectively. RIPC comprised of three cycles of cuff inflation each lasting 5 min in duration followed by 5‐min period of cuff deflation. A manual BP cuff (HEINE Gamma XXL LF^®^, HEINE Optotechnik, Herrsching, Germany) was used for upper limb RIPC while an automated rapid cuff inflator (Hokanson E20, D.E. Hokanson, Inc., Bellevue, WA, USA) with an 8‐inch‐wide cuff was used to induce lower limb RIPC.

### Blood samples

Mixed capillary blood was obtained via finger prick (for the upper limbs protocols) and via toe prick (for the lower limbs protocols) prior to RIPC (95 *μ*L of capillary blood was drawn in heparinized capillary tubes) at the time points shown in the protocol (Fig. 1) and analyzed for blood pH, partial pressures of O_2_ (pO_2_) and CO_2_ (pCO_2_), bicarbonate (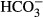 ), base excess (BE), oxygen saturation (sO_2_), and blood lactate levels using a handheld blood chemistry analyzer (Abbott Diagnostics iStat 1 analyzer [iStat Corp, East Windsor, NJ, USA] and i‐STAT CG4+ cartridges [Abbott Laboratories Ltd., Berkshire, UK]).

### Other measurements

Presence or absence of pulsatile vascular flow was assessed during each cycle using a Doppler ultrasound device (Huntleigh dopplex MD2 device, Huntleigh Healthcare Ltd., Cardiff, UK). Subjective pain scores reported by volunteers were also assessed by using a standard (0–10) numerical rating scale (NRS) (Ferreira‐Valente et al. 2011). These were obtained at baseline, at the end of each inflation–deflation during RIPC, as well as during recovery.

### Statistical analysis

D'Agostino & Pearson omnibus normality test was carried out to ensure that measurements were normally distributed. Two way repeated measures ANOVA analyses (treatment × time) was carried out to assess significance of changes in various blood gas parameters as well as in the severity of pain reported by volunteers with RIPC in the upper and lower limb using various cuff inflation pressures. All the statistical analyses were performed with Graphpad Prism 6 (GraphPad Software, Inc., La Jolla, CA, USA). Alpha was set at 0.05 level. All values are expressed as Mean ± Standard Error, unless specified.

## Results

The mean height of the volunteers was 177.4+1.6 cm, the mean weight was 77.6 ± 4.3 kg and the mean BMI was 24.6 ± 1.3 kg/m^2^. Mean arm circumference was 31.6 ± 1.2 cm, whereas the mean mid‐thigh circumference was 51.8 ± 1.3 cm. This difference in the mean circumference between thigh and arm was statistically significant (*P* < 0.01).

The mean blood pressures recorded at baseline, at the end of RIPC and at 10 min into recovery post RIPC are summarized in Table 1. No significant changes were noted in the mean systolic or diastolic BP when measured at the end of RIPC compared with the baseline values.

**Table 1.** Blood pressures values (mean ± SE) recorded in volunteers at baseline, immediately following RIPC (30 min from start of RIPC protocol) and after 10 min of recovery post RIPC No significant changes observed in variables across time or between conditions

RIPC protocol evaluated	Measured blood pressures (mm of Hg)	Baseline	Post RIPC (0 min)	Post RIPC (+10 min)
140 mmHg (UL)	Systolic	131.1 ± 5.5	126.9 ± 5.2	128.2 ± 5.5
	Diastolic	77.6 ± 3.3	78.0 ± 4.6	78.2 ± 4.8
140 mmHg (LL)	Systolic	127.0 ± 3.9	126.2 ± 5.6	128.3 ± 5.0
	Diastolic	76.8 ± 4.0	82.3 ± 5.2	80.4 ± 4.7
160 mmHg (UL)	Systolic	130.7 ± 5.4	128.6 ± 3.6	129.6 ± 3.0
	Diastolic	75.8 ± 4.2	76.1 ± 4.2	74.58 ± 4.0
160 mmHg (LL)	Systolic	129.1 ± 4.2	130.8 ± 4.8	127.8 ± 5.6
	Diastolic	76.7 ± 4.9	82.4 ± 4.9	80.1 ± 4.4
180 mmHg (UL)	Systolic	127.9 ± 3.1	124.8 ± 4.1	126.7 ± 4.8
	Diastolic	73.3 ± 3.2	74.0 ± 3.8	73.4 ± 4.2
180 mmHg (LL)	Systolic	125.0 ± 2.7	126.8 ± 4.0	123.7 ± 2.9
	Diastolic	74.6 ± 2.3	75.7 ± 2.8	75.8 ± 2.1

### Arterial blood flow

**Figure 2.**
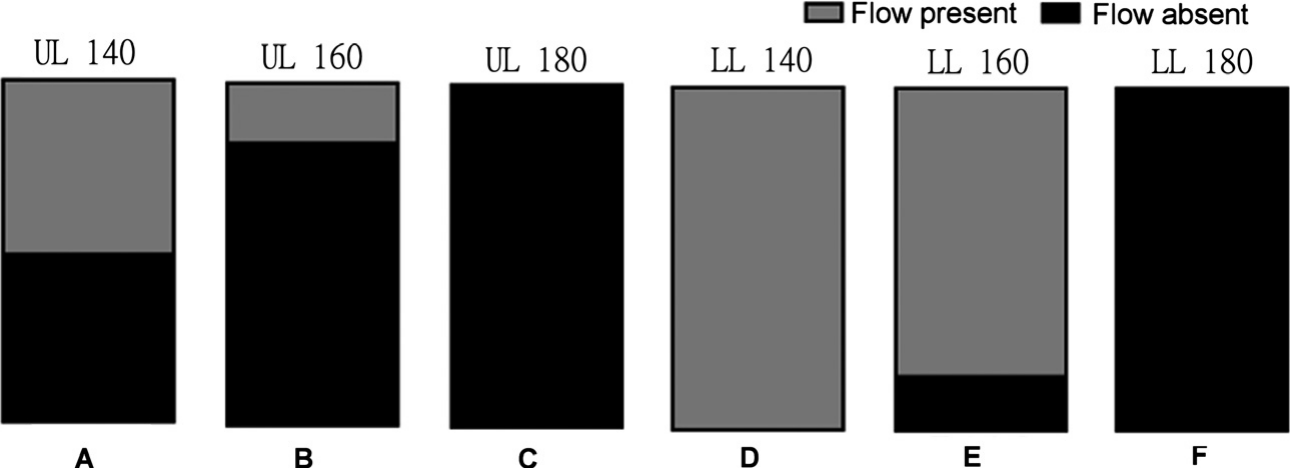
Illustration of the fraction of the total number volunteers (*n *=**6) in whom arterial blood flow was occluded (shown in black) at each cuff inflation pressure per limb. (A) 140 mmHg (UL); (B) 160 mmHg (UL); (C) 180 mmHg (UL); (D) 140 mmHg (LL); (E) 160 mmHg (LL); (F) 180 mmHg (LL); UL, upper limb, LL, lower limb.

Cuff inflation pressure of 140 mmHg (Upper limb), caused a cessation of pulsatile arterial blood flow in three of six volunteers during all three RIPC cycles. This was identified through the loss of Doppler signal in the radial artery in the corresponding limb, distal to the site of cuff inflation. At 160 mmHg cuff inflation pressure (Upper Limb), arterial blood flow was successfully occluded during all occlusions in five of six volunteers. At an inflation pressure of 180 mmHg in the upper limb, arterial blood flow was occluded in all three cuff inflations and in all individuals (Fig. 2).

In the upper limb complete cessation of blood flow in all of the chosen RIPC cuff inflations was reliably achieved when the occlusion pressure was 30 mm of Hg or more higher than the subjects mean resting systolic pressure.

In the lower limb, a cuff inflation pressure of 140 mmHg did not occlude arterial flow in any of the volunteers. Presence or absence of pulsatile flow was assessed in the posterior tibial artery in the lower limb. At 160 mmHg, arterial flow was occluded during all three cuff inflations in only one out of six volunteers. At 180 mmHg cuff inflation pressure, all cuff inflations led to cessation of arterial flow in the limb (Fig. 2).

In the lower limb, a cuff inflation pressure approximately 50–55 mmHg higher than the resting mean systolic blood pressure was required to reliably occlude arterial flow distally in the limb.

### Physiological changes in the limb during RIPC

#### Effect of RIPC on blood oxygen levels

Blood oxygenation was assessed from changes in partial pressure of oxygen (pO_2_) and oxygen saturation (sO_2_).

**Figure 3.**
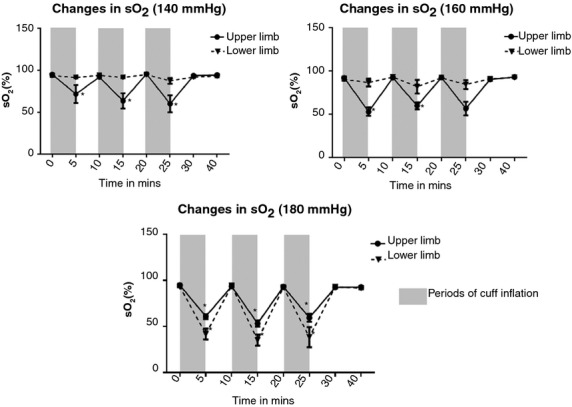
Changes in the capillary blood oxygen saturation with varying cuff inflation pressures per limb. While significant changes were noted in the mean capillary oxygen saturation levels at all cuff inflation pressures applied to the upper limb, in the lower limb only the highest cuff inflation pressure used (180 mmHg) led to significant changes.

A significant reduction was noted in the mean sO_2_ (%) in the upper limb with each of the three cuff inflations during all three cycles of RIPC at all the cuff inflation pressures (140, 160, and 180 mmHg) (*P* < 0.01). In the lower limb, only the highest cuff inflation pressure of 180 mmHg led to a significant reduction in the mean sO_2_ (%) level in the limb compared with the baseline (Table 2; Fig. 3). In all the cases where there was a significant reduction in sO_2_ during cuff inflation, the mean sO_2_ (%) levels returned back to baseline after 5 min of reperfusion of the limb after the cuff was deflated. Similarly, significant reduction in the pO_2_ was noted with each cuff inflation during the three cycles of RIPC at each of the three cuff inflation pressures (140, 160, and 180 mmHg) in the upper limb (*P* < 0.01) (Table 2). In the lower limb only the higher cuff inflation pressure of 180 mmHg was able to cause a significant reduction in the pO2 levels. After 5 min of reperfusion of the limb with the cuff deflated, the pO_2_ levels returned back to the baseline in all cases. These changes in sO_2_ and pO_2_ over the duration of the three cycles of RIPC varied significantly based on the cuff inflation pressure and limb used (*P* < 0.01). While there were comparable significant changes in sO_2_ and pO_2_ levels in both the upper and lower limbs with cuff inflation at 180 mmHg pressure, cuff inflation at lower pressures of 140 and 160 mmHg led to significant changes in the upper limb but not in the lower limb.

**Table 2.** Summary of changes in capillary arterial blood and pain associated with three cycles of RIPC using either 140, 160, and 180 mmHg cuff inflation pressure on the upper and lower limbs; UL = Upper Limb, LL = Lower Limb; 140/160/180 = cuff inflation pressure used for RIPC; *indicates significant changes over time course of RIPC intervention

Time (in minutes)→		Baseline	5	10	15	20	25	30	40
sO_2_ (%)	UL 140*	95.2 ± 0.8	71.8 ± 10.7	92.2 ± 1.0	63.7 ± 9.2	95.8 ± 0.7	60.2 ± 10.1	93.8 ± 0.7	94.5 ± 0.7
	UL 160*	92.3 ± 1.6	53.2 ± 5.0	92.3 ± 0.6	60.0 ± 4.1	92.7 ± 0.6	56.8 ± 7.9	90.5 ± 1.8	93.2 ± 1.3
	UL 180*	94.8 ± 1.3	61.0 ± 2.8	93.7 ± 0.8	53.3 ± 3.5	93.3 ± 1.1	59.5 ± 4.3	92.5 ± 1.2	92.8 ± 1.2
	LL 140	93.8 ± 1.4	91.7 ± 1.2	94.2 ± 0.8	91.7 ± 1.0	95.0 ± 1.2	87.7 ± 3.5	92.2 ± 1.1	93.3 ± 1.5
	LL 160	90.5 ± 1.6	86.8 ± 4.5	93.2 ± 0.3	82.0 ± 7.9	92.3 ± 0.8	84.5 ± 5.3	91.2 ± 1.1	92.8 ± 0.9
	LL 180*	93.2 ± 0.6	41.8 ± 6.0	94.3 ± 1.2	35.3 ± 6.0	92.5 ± 1.7	38.5 ± 10.9	93.2 ± 0.9	91.7 ± 1.2
pO_2_ (kPa)	UL 140*	10.4 ± 0.7	6.3 ± 1.2	8.7 ± 0.4	5.1 ± 0.8	10.9 ± 0.7	4.6 ± 0.6	9.1 ± 0.4	9.7 ± 0.5
	UL 160*	8.9 ± 0.6	4.0 ± 0.3	8.7 ± 0.3	4.4 ± 0.3	8.8 ± 0.3	4.4 ± 0.5	8.2 ± 0.6	9.0 ± 0.5
	UL 180*	11.8 ± 2.6	4.5 ± 0.2	9.3 ± 0.4	4.0 ± 0.2	9.2 ± 0.6	4.4 ± 0.3	9.0 ± 0.5	9.1 ± 0.4
	LL 140	8.8 ± 0.6	8.4 ± 0.4	9.4 ± 0.4	8.4 ± 0.4	11.4 ± 1.9	7.9 ± 0.8	8.6 ± 0.4	9.2 ± 0.6
	LL 160	8.1 ± 0.4	7.9 ± 0.9	8.9 ± 0.2	7.3 ± 1.0	8.5 ± 0.2	7.3 ± 0.8	8.1 ± 0.4	8.7 ± 0.4
	LL 180*	8.9 ± 0.4	3.2 ± 0.3	10.0 ± 0.9	2.9 ± 0.4	9.4 ± 1.0	3.3 ± 0.8	8.9 ± 0.4	8.5 ± 0.4
pCO_2_ (kPa)	UL 140*	5.1 ± 0.2	5.5 ± 0.4	5.3 ± 0.1	5.9 ± 0.1	5.1 ± 0.2	6.1 ± 0.3	5.4 ± 0.1	5.2 ± 0.1
	UL 160*	5.2 ± 0.1	6.3 ± 0.0	5.3 ± 0.1	6.1 ± 0.1	5.3 ± 0.1	6.1 ± 0.2	5.2 ± 0.1	5.3 ± 0.2
	UL 180*	4.8 ± 0.3	5.8 ± 0.1	5.2 ± 0.2	6.0 ± 0.2	5.2 ± 0.1	6.0 ± 0.2	5.4 ± 0.1	5.3 ± 0.2
	LL 140	5.3 ± 0.2	5.0 ± 0.1	5.0 ± 0.2	5.1 ± 0.1	4.7 ± 0.3	5.1 ± 0.3	5.1 ± 0.2	4.9 ± 0.3
	LL 160	5.2 ± 0.1	5.2 ± 0.1	5.1 ± 0.1	5.2 ± 0.2	5.1 ± 0.1	5.1 ± 0.1	5.1 ± 0.2	4.9 ± 0.2
	LL 180*	5.2 ± 0.2	5.5 ± 0.3	5.1 ± 0.2	5.8 ± 0.3	5.1 ± 0.1	5.9 ± 0.3	4.9 ± 0.2	5.2 ± 0.1
pH	UL 140*	7.41 ± 0.01	7.39 ± 0.02	7.41 ± 0.01	7.36 ± 0.01	7.43 ± 0.02	7.36 ± 0.02	7.41 ± 0.01	7.41 ± 0.00
	UL 160*	7.41 ± 0.01	7.34 ± 0.02	7.41 ± 0.01	7.36 ± 0.01	7.42 ± 0.01	7.36 ± 0.02	7.42 ± 0.01	7.42 ± 0.01
	UL 180*	7.43 ± 0.02	7.35 ± 0.01	7.41 ± 0.01	7.35 ± 0.01	7.41 ± 0.01	7.36 ± 0.01	7.40 ± 0.01	7.40 ± 0.01
	LL 140	7.41 ± 0.02	7.43 ± 0.01	7.43 ± 0.01	7.40 ± 0.01	7.43 ± 0.02	7.41 ± 0.03	7.41 ± 0.01	7.43 ± 0.02
	LL 160	7.40 ± 0.01	7.41 ± 0.01	7.42 ± 0.01	7.41 ± 0.02	7.41 ± 0.01	7.41 ± 0.01	7.42 ± 0.01	7.42 ± 0.01
	LL180	7.41 ± 0.01	7.39 ± 0.02	7.42 ± 0.02	7.37 ± 0.02	7.42 ± 0.02	7.38 ± 0.02	7.43 ± 0.01	7.41 ± 0.01
HCO_3_ (mmol/L)	UL 140	24.4 ± 0.5	24.3 ± 0.8	25.0 ± 0.4	25.3 ± 0.7	25.4 ± 0.5	25.7 ± 0.5	25.1 ± 0.5	25.0 ± 0.5
	UL 160	24.9 ± 0.7	25.9 ± 0.9	25.0 ± 0.6	25.8 ± 0.8	25.7 ± 0.7	26.2 ± 0.6	25.3 ± 0.5	25.5 ± 0.8
	UL 180	24.1 ± 0.5	23.9 ± 0.4	24.8 ± 0.7	24.5 ± 0.8	25.0 ± 0.7	25.5 ± 0.6	25.3 ± 0.6	24.9 ± 0.7
	LL 140	25.1 ± 0.3	24.7 ± 0.7	24.3 ± 0.8	24.2 ± 0.5	23.0 ± 0.6	24.3 ± 0.3	24.3 ± 0.6	24.2 ± 0.5
	LL 160	24.5 ± 0.9	24.4 ± 0.8	24.6 ± 0.8	24.6 ± 0.8	24.3 ± 0.7	24.2 ± 0.5	24.6 ± 0.5	24.1 ± 0.9
	LL 180	24.7 ± 0.7	25.0 ± 0.9	24.6 ± 0.8	25.3 ± 0.7	24.9 ± 0.8	25.7 ± 0.78	24.6 ± 0.6	24.8 ± 0.7
BE (mmol/L)	UL 140	−0.3 ± 0.3	−0.7 ± 0.7	0.3 ± 0.4	−0.2 ± 0.9	1.0 ± 0.8	0.3 ± 0.5	0.5 ± 0.6	0.3 ± 0.5
	UL 160	0.3 ± 0.8	0.2 ± 1.1	0.3 ± 0.8	0.2 ± 1.0	1.2 ± 0.8	1.0 ± 0.9	0.5 ± 0.6	1.2 ± 0.9
	UL 180	−0.2 ± 0.8	−1.5 ± 0.5	0.3 ± 0.8	−0.7 ± 1.1	0.3 ± 0.7	0.0 ± 0.6	0.5 ± 0.6	0.0 ± 0.8
	LL 140	0.3 ± 0.4	0.3 ± 0.8	0.2 ± 0.8	−0.3 ± 0.6	−1.3 ± 0.61	−0.3 ± 0.7	−0.3 ± 0.8	0.0 ± 0.5
	LL 160	−0.3 ± 1.1	−0.3 ± 0.9	0.2 ± 0.9	−0.2 ± 1.1	−0.3 ± 0.8	−0.5 ± 0.5	0.0 ± 0.5	−0.3 ± 1.0
	LL 180	0.0 ± 0.7	−0.2 ± 1.0	0.2 ± 0.9	0.0 ± 0.5	0.7 ± 1.0	0.7 ± 0.8	0.5 ± 0.6	0.00.9
Lactate (mmol/L)	UL 140*	1.4 ± 0.3	2.3 ± 0.1	1.3 ± 0.3	1.9 ± 0.1	1.1 ± 0.2	2.0 ± 0.3	1.1 ± 0.2	1.0 ± 0.2
	UL 160*	1.3 ± 0.2	2.1 ± 0.2	0.9 ± 0.1	1.8 ± 0.2	0.8 ± 0.1	2.0 ± 0.3	0.9 ± 0.1	0.8 ± 0.2
	UL 180*	1.4 ± 0.2	2.5 ± 0.3	1.2 ± 0.3	2.6 ± 0.3	1.1 ± 0.3	2.5 ± 0.3	1.1 ± 0.3	1.1 ± 0.2
	LL 140	1.3 ± 0.2	1.2 ± 0.2	1.0 ± 0.1	1.1 ± 0.2	1.0 ± 0.2	1.3 ± 0.2	1.0 ± 0.1	1.0 ± 0.1
	LL 160	1.2 ± 0.3	1.1 ± 0.3	0.8 ± 0.1	1.2 ± 0.2	0.9 ± 0.2	1.1 ± 0.2	1.0 ± 0.2	0.9 ± 0.2
	LL 180*	1.1 ± 0.1	1.9 ± 0.2	0.9 ± 0.1	2.0 ± 0.2	1.0 ± 0.2	1.9 ± 0.3	0.9 ± 0.1	0.8 ± 0.1
Pain scores (0–10)	UL 140*	0.0 ± 0.0	4 ± 0.5	0.0 ± 0.0	4 ± 0.4	0.0 ± 0.0	3 ± 0.6	0.0 ± 0.0	0.0 ± 0.0
	UL 160*	0.0 ± 0.0	4 ± 0.5	0.3 ± 0.3	4 ± 0.7	0.5 ± 0.5	4 ± 0.6	0.3 ± 0.3	0.0 ± 0.0
	UL 180*	0.0 ± 0.0	4 ± 0.7	0.0 ± 0.0	5 ± 0.4	0.0 ± 0.0	5 ± 0.6	0.0 ± 0.0	0.0 ± 0.0
	LL 140*	0.0 ± 0.0	5 ± 0.7	0.0 ± 0.0	4 ± 0.6	0.0 ± 0.0	3 ± 0.6	0.0 ± 0.0	0.0 ± 0.0
	LL 160*	0.0 ± 0.0	6 ± 0.8	0.0 ± 0.0	6 ± 0.6	0.0 ± 0.0	5 ± 1	0.3 ± 0.2	0.0 ± 0.0
	LL 180*	0.0 ± 0.0	7 ± 0.4	0.0 ± 0.0	6 ± 0.8	0.0 ± 0.0	6 ± 0.3	0.0 ± 0.0	0.0 ± 0.0

#### Effect of RIPC on blood pCO_2_

**Figure 4.**
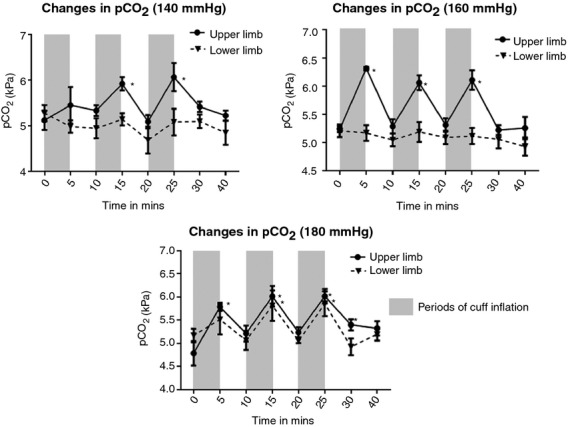
Significant rise (from baseline) in pCO_2_ is noted with cuff inflation in the upper limb at all pressures (*P *<**0.01). For lower limb, values reach significance at 180 mmHg cuff inflation pressure only.

In the upper limb, significant increases were noted in the pCO_2_ levels at all the cuff inflation pressures assessed (Table 2; Fig. 4). At 140 mmHg, there was a significant increase in the capillary blood pCO_2_ levels in the upper limb at the end of the second and the third cuff inflation only. At 160 and 180 mmHg cuff inflation pressures (UL), a significant increase in the pCO_2_ levels was observed in all cycles of RIPC (*P* < 0.01). At the end of 5 min of the cuff deflation, pCO_2_ levels returned back to baseline with the exception of the third cuff inflation at 180 mmHg, where 10‐min recovery time was required before pCO_2_ levels returned to baseline. In comparison, no significant changes were noted in the pCO_2_ levels at 140 and 160 mmHg cuff inflations, in the lower limb. At 180 mmHg there was a progressive rise in pCO_2_ levels with successive cuff inflations, values reaching significance during the second and third cuff inflation (*P* < 0.01). In the lower limb, the pCO_2_ levels recovered back to the baseline at the end of 5 min of cuff deflation. Significant effects were observed for “inflation pressure” and “limb” for pCO2 levels (*P* = 0.0003). In the upper limb, all three cuff inflation pressures led to significant increase in the mean pCO_2_ levels recorded at the end of 5 min of cuff inflation, while conversely, only the highest cuff inflation pressure was able to induce significant increase in capillary arterial levels of pCO_2_ in the lower limb.

#### Effect of RIPC on pH, 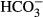 levels and Base Excess

With regard to changes in pH, there was a significant decrease in the pH of the capillary arterial blood with each cuff inflation at each of the three cuff inflations pressures apart from the first cuff inflation in the upper limb at 140 mmHg (*P* < 0.01). Interestingly, none of the cuff inflations in the lower limb at any of the pressures (140, 160, or 180 mmHg) were able to change the pH significantly from baseline. There was no significant change in the 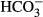 levels (*P* > 0.05) or Base excess (*P* = 0.09) in the capillary arterial blood with cuff inflations at any of the three cuff inflation pressures assessed in either the upper or the lower limb.

#### Effect of RIPC on lactate levels

**Figure 5.**
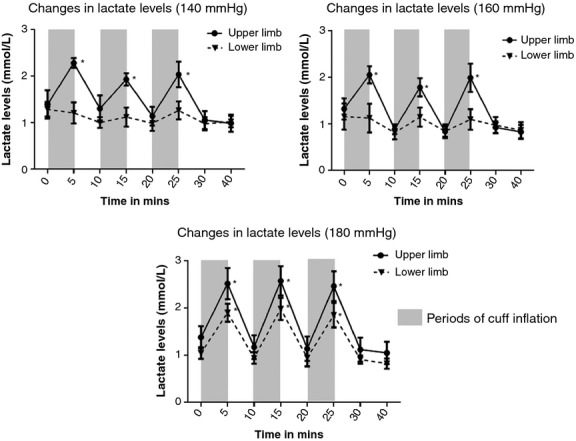
In the upper limb, cuff inflation at 140, 160 and 180 mmHg during all three cycles of RIPC led to cyclical rise in the capillary arterial lactate levels, which returned to baseline by the end of 5 min of cuff deflation. In the lower limb, significant cyclical rise and fall in capillary arterial lactate levels was only noted at a cuff inflation pressure of 180 mmHg.

There was a significant rise in the mean capillary arterial blood lactate levels with each of the three cuff inflations at 140, 160, and 180 mmHg cuff inflation pressures (*P* < 0.01) (Table 2). In all these cases, the mean lactate levels returned back to baseline levels at the end of 5 min of cuff deflation (Table 2; Fig. 5). Though there was a cyclical rise and fall in the mean lactate levels with the three cycles of RIPC, there was no cumulative increase in the lactate levels with successive cycles of RIPC. In the lower limb, similar significant increase in the mean lactate levels was only noted at 180 mmHg cuff inflation pressure (*P* < 0.01).

### Perception of pain associated with RIPC using different cuff inflation pressures

**Figure 6.**
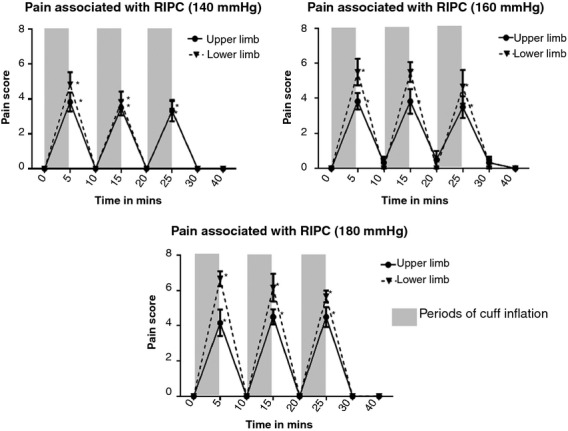
All the cuff inflations in the upper and lower limb at 140, 160 and 180 mmHg were associated with significant pain and discomfort (measured on a standard 0–10 Numeric Rating Pain Scale). At 160 and 180 mmHg cuff inflation pressures, the pain/discomfort associated with RIPC was significantly higher in the lower limb compared with the upper limb in the first two cycles of RIPC.

In both the upper and lower limbs, there was a significant increase in the pain score from baseline with all three cuff inflation pressures used for RIPC (*P* < 0.01) (Table 2; Fig. 6). The mean pain score was not significantly different in any of the cuff inflations between the upper and the lower limb at 140 mmHg cuff inflation. However, at both 160 and 180 mmHg cuff inflation pressure, the pain associated with the cuff inflation was significantly more in the lower limb compared with the upper limb in the first two cycles of RIPC (*P* < 0.01). In the third cycle of RIPC, there was no significant difference in the pain associated with cuff inflations even at the higher 160 and 180 mmHg inflation pressures.

## Discussion

The aim of this study was to assess the dose–response relationship of an increase in the cuff inflation pressure used for RIPC with the physiological changes that occur in the upper and lower limb. In addition, the ability of various cuff inflation pressures to cease pulsatile blood flow in the upper and lower limb as well as the pain/discomfort associated with the respective cuff inflation pressures was investigated. Results showed that cessation of arterial blood flow in the upper limb was achieved in all the cases in our study when the cuff inflation pressure was ~30 mmHg higher than the resting systolic blood pressure. In comparison, in the lower limb arterial flow was only reliably occluded during all RIPC cuff inflations at 180 mmHg which was ~55 mmHg higher than the mean resting systolic pressure. Thus a higher cuff inflation pressure was required to terminate pulsatile blood flow in the lower limb compared with the upper limb. Analysis of physiological changes in the limb during ischemia demonstrated a reduction in sO_2_ (%), pO_2_ levels and pH in the limb as well as an increase in the levels of CO_2_ and lactate, but without any change in the base excess or 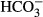 levels at all cuff inflation pressures in the upper limb. However, in the lower limb, these changes were present to a significant level only at the highest 180 mmHg cuff inflation pressure. Even the highest cuff inflation pressure used (180 mmHg) was not sufficient to cause significant changes in pH in the lower limb. All the changes that were seen during cuff inflation reverted back to baseline with 5 min of limb reperfusion when the cuff was deflated. Therefore, ischemic changes were more easily induced at lower cuff inflation pressures in the upper limb compared with the lower limb. One obvious reason could be the vast difference in the mean limb circumference between the upper and lower limb. The mean arm circumference in our study was 31.6 ± 1.2 cm which was significantly lower than the mean lower limb circumference 51.8 ± 1.3 cm. Hence, in the upper limb the cuff occlusion was resisted by a smaller bulk of muscle/tissue compared with the lower limb.

RIPC was better tolerated in the upper limb compared with the lower limb at higher cuff inflation pressures. At the lowest cuff inflation pressure studied (140 mmHg), there was no significant difference in the pain scores reported by the volunteers during any of the three cycles. However, in the first two cycles of RIPC at the higher 160 and 180 mmHg cuff inflation pressures, pain/discomfort associated with RIPC was significantly higher in the lower limb compared with RIPC in the upper limb. Interestingly, in the third cycles of RIPC at these cuff inflations pressures, there was no longer a significant difference in the pain associated with RIPC as there was a progressive reduction in the pain/discomfort perceived by the volunteers with consecutive cycles of RIPC in the lower limb. This is an intriguing finding, though the mechanisms underlying this gradual reduction in the pain/discomfort associated with RIPC in nonanesthetized subjects remains to be investigated. Overall, the present results suggest that limb ischemia for RIPC can be induced in the upper limb at lower pressures compared with the lower limb and that this is better tolerated. However, whether protection against IR injury in the target organs or improvement in athletic performance can also be induced at lower cuff inflation pressures in the upper limb compared with the lower limb remains to be studied. This would be important to study as clearly cuff inflations were better tolerated in the upper limb compared with the lower limb, apart from the extremely low cuff inflation pressure of 140 mmHg which was equally well tolerated in the upper and lower limb.

However, these results do highlight that the physiological responses and tolerability of RIPC vary according to the limb and the blood pressure cuff inflation pressure used; thereby demonstrating a need to take these factors into account in designing future RIPC clinical trials. Clearly, further studies are required to directly compare the upper and lower limbs in their ability to deliver the benefits from RIPC. Future research into this subject in a target population using appropriate protection end‐points will help establish effective RIPC protocols that are also the most tolerated.

## Conflict of Interest

The authors have no conflicts of interest to declare.
